# Expression patterns of hair-related keratins and epithelial keratins in onychopapilloma: The significance of clarifying the origin of onychopapilloma

**DOI:** 10.3389/fmed.2022.1059624

**Published:** 2022-11-22

**Authors:** Mengxi Liu, Fang Li, Xiaoqing Wang, Zhenru Liu, Hoi Shiwn Wong, Yuan Zhou, Daguang Wang

**Affiliations:** ^1^Department of Dermatology, The First Affiliated Hospital of Nanjing Medical University, Nanjing, China; ^2^Department of Dermatology, Suzhou Municipal Hospital East Area, The Affiliated Suzhou Hospital of Nanjing Medical University, Suzhou, China; ^3^Institute of Dermatology, Chinese Academy of Medical Sciences and Peking Union Medical College, Nanjing, China

**Keywords:** onychopapilloma, nail, hair-related keratins, epithelial keratins, immunohistochemical study

## Abstract

**Background:**

Onychopapilloma is generally recognized as a benign tumor of the nail bed and distal matrix. However, the origin of onychopapilloma has not been explained yet.

**Objective:**

To clarify the origin of onychopapilloma, we detected the expression patterns of hair-related keratins and epithelial keratins, which are expressed specifically in the nail unit.

**Materials and methods:**

The clinical and histopathologic features of 11 patients with onychopapilloma were analyzed, and the expression patterns of hair-related and epithelial keratins were detected.

**Results:**

Histologically, all subjects showed acanthosis, papillomatosis and matrix metaplasia within the nail bed. Immunohistochemically, the expression pattern of keratins in our standard nail unit was consistent with previous reports. “Nail matrix-related keratins” HK31, HK34, HK85, and HK86 were only expressed in the nail matrix, and “Nail bed-related keratins” HK75 and K6/K16 were only expressed in the nail bed. However, in onychopapilloma, whether adjacent to the matrix or in the distal nail bed, all cases were positive for nail bed-related keratins and HK31 but negative for other nail matrix-related keratins.

**Conclusion:**

Our study suggests that onychopapilloma may originate from the nail bed rather than the nail matrix. Furthermore, the expression of nail bed-related keratins and HK31 could be used as diagnostic markers of onychopapilloma.

## Introduction

Onychopapilloma is recognized as a benign tumor of the nail bed and distal nail matrix. It was first described as “localized multinucleate distal subungual keratosis” by Baran and Perrin (1), and the term “onychopapilloma” was proposed in 2000 (2). It usually occurs on the thumbs of adults (3). Although Kim et al. conjectured that onychopapilloma could be neoplastic hyperplasia of the nail bed epithelium or reactive hyperplasia of the nail bed epithelium due to chronic irritation, trauma or other inflammatory nail diseases (4), there is no definite evidence of this. Whether the onychopapilloma originates from the nail bed, the nail matrix, or both are discussed in this study.

The nail bed is different from the nail matrix in histology and embryology, and there is no transformation between the developed nail bed and the nail matrix. Anatomically, the nail unit is usually recognized as an equivalent of the follicle. Some authors claimed that the nail bed is equivalent to the outer root sheath of the follicle and that the nail matrix is comparable to the hair matrix (5, 6). In recent studies, the expression profiles of hair-related and epithelial keratins have been determined in the human nail unit (6–9). The nail matrix was the only site of expression of hair-related keratins, including HK31, HK34, HK85, and HK86, which could not be detected in the nail bed and hyponychium. HK75, K6, and K16 were explicitly expressed in the nail bed. The unique expression pattern of hair-related keratins and epithelial keratins in the nail unit could be used to identify the differentiation of nail matrix epithelium and nail bed epithelium. However, a comprehensive immunohistochemical analysis of keratin expression in onychopapilloma has not been performed. To define the origin of onychopapilloma, we analyzed the pathologic characteristics of this disease and the expression patterns of hair-related keratins and epithelial keratins in the involved tissue. Furthermore, we hope to search for special markers of onychopapilloma.

## Materials and methods

### Tissue samples

All subjects gave their informed consent for inclusion before they participated in the study. The study was conducted in accordance with the Declaration of Helsinki, and the protocol was approved by the Ethics Committee of the First Affiliated Hospital of Nanjing Medical University (IRB-GL1-AF05; 2021-NT-32).

Surgically resected specimens from 11 cases with onychopapilloma treated between August 2019 and May 2021 at the Department of Dermatology in the First Affiliated Hospital of Nanjing Medical University were used for this study. The following features were recorded: age, sex, affected nail(s) location, and clinical presentations. We performed two types of biopsies according to the actual situation: (1) incision biopsies extending from the distal nail matrix to the hyponychium, including the nail matrix, the nail bed, the proximal nail fold and the hyponychium; and (2) longitudinal biopsies, in which the scope is the same as the biopsy after nail avulsion. All samples were fixed in 4% buffered formalin and embedded in paraffin. We selected seven standard nails as the control (normal nail unit tissue specimen around the skin lesion after nail matrix nevi excision).

### Immunohistochemistry

All antibodies were first assayed on formalin-fixed and paraffin-embedded scalp sections to demonstrate their correct reactivity relative. The following hair-related keratins and epithelial keratins were used for the immunohistochemical analysis: HK31 (1:1,000; Thermo Fisher, Massachusetts, USA), HK34 (1:400; Progen), HK 85 (1:400; Progen, Heidelberg, Germany), HK 86 (1:400; Progen), HK75 (1:200; Thermo Fisher) and K6/K16 (1:300; Thermo Fisher). SP methods were employed to perform the immunohistochemical staining, and the steps were performed according to the standard protocol.

## Results

### Clinical presentations

**FIGURE 1:**
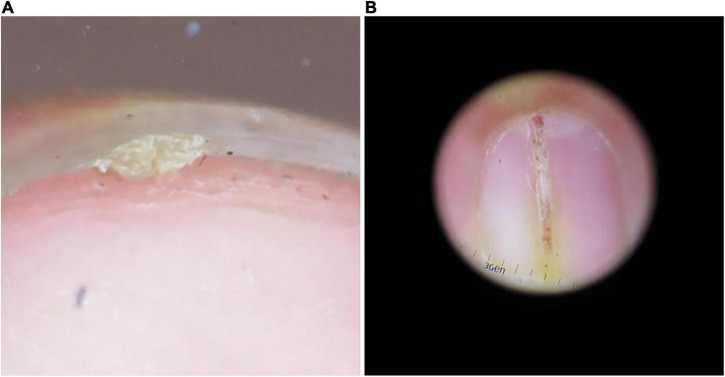
Common clinical presentations of onychopapilloma. **(A)** Subungual keratotic mass. **(B)** Longitudinal erythronychia.

Eleven onychopapilloma cases were identified (Table 1), including six females and five males ranging from 13 to 54 (mean, 30.7) years of age. The thumb was the most affected digit (*n* = 6). Subungual hyperkeratosis and a longitudinal band were the most common clinical presentations (Figure 1). In all cases, the longitudinal band corresponded to the keratotic mass under the nail plate. Splinter hemorrhages with longitudinal erythronychia were observed in 2 cases, and V-shaped notches and splitting of the distal nail plate were observed in another 2 cases.

**TABLE 1:** Clinical presentations of onychopapilloma.

Clinical presentations	Total cases	Subungual hyperkeratosis	Splinter hemorrhage	Distal V-shaped notch and split
Longitudinal erythronychia	6	6	2	0
Longitudinal melanonychia	3	3	0	1
Longitudinal leukonychia	2	2	0	1
Total cases	11	11	2	2

### Pathology

**FIGURE 2:**
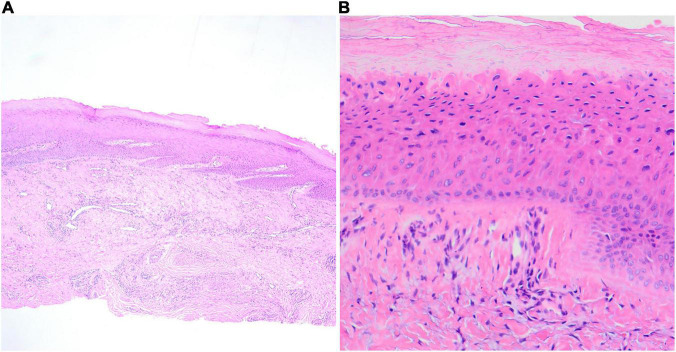
Longitudinal sections; the pathological features of onychopapilloma. **(A)** Papillomatosis of the distal nail bed epithelium (HE, ×100). **(B)** The scanning magnification shows the upper layers of the nail bed contains cells resembling those of the keratogenous zone of the matrix (HE, ×400).

In all cases, the most obvious histological presentation of specimens was papillomatosis with or without acanthosis of the distal nail bed epithelium (Figure 2). Most cases showed focal proliferation of epithelial cells with abundant eosinophilic cytoplasm in the upper layers of the nail bed epithelium (matrix metaplasia), which resembles the keratogenous zone of the matrix. In some cases, dyskeratosis cells could be observed in the superficial layer of the nail bed epithelium. Subungual hyperkeratosis could be seen under the distal nail plate in the longitudinal biopsy specimens. However, there was no obvious abnormality in the nail matrix in 11 cases.

### Immunohistochemical study

The immunohistochemical results showed the same expression pattern of hair-related keratins and epithelial keratins in the standard nail unit as in previous reports, which was similar to their sequential expression in the hair follicle cortex (Table 2). HK31, HK34, HK85, and HK86 were consecutively expressed in the keratogenous zone of the distal nail matrix. The expression of HK75 was only observed in the nail bed. K6/K16 was expressed in the apical nail matrix and the nail bed but still not in the distal nail matrix.

**TABLE 2:** Hair-related keratins and epithelial keratins expression in the normal nail unit and onychopapilloma.

	HK31	HK34	HK85	HK86	HK75	K6/K16
Normal nail matrix	+	+	+	+	–	–
Normal nail bed	–	–	–	–	+	+
Onychopapilloma	+	–	–	–	+	+

**FIGURE 3:**
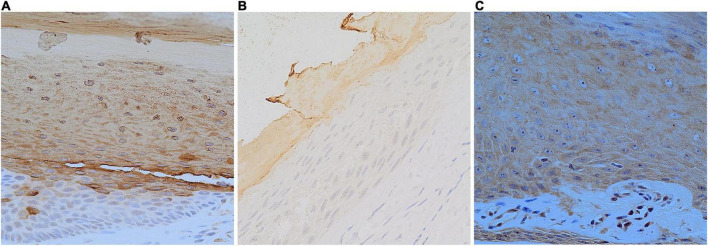
Longitudinal sections; expression of HK31 in the normal nail unit and onychopapilloma. **(A)** Normal nail matrix (DAB staining, SP, ×400). **(B)** Normal nail bed (DAB staining, SP, ×400). **(C)** Nail bed of onychopapilloma (DAB staining, SP, ×400).

**FIGURE 4:**
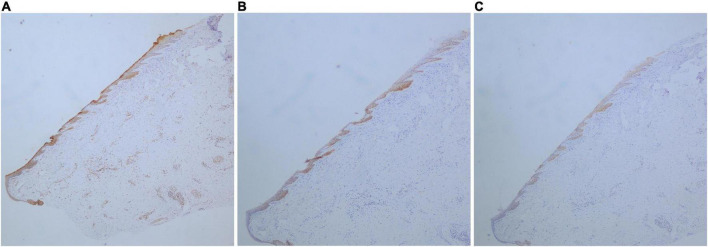
Longitudinal sections; expression of HK31, HK75, and K6/K16 in the nail bed of the onychopapilloma. **(A)** Expression of HK31 (DAB staining, SP, ×40). **(B)** Expression of HK75 (DAB staining, SP, ×40). **(C)** Expression of K6/K16 (DAB staining, SP, ×40).

Of all the onychopapilloma cases, hair-related keratins, including HK34, HK85, and HK86, still showed negative expression in the nail bed (Table 3). However, all onychopapilloma cases showed positive expression of HK31 (Figure 3), whether adjacent to the matrix or in the distal nail bed (Figure 4). As expected, “nail bed-related keratins” HK75 and K6/K16 were positively expressed in onychopapilloma, especially papillomatosis with acanthosis of the distal nail bed epithelium (Table 3).

**TABLE 3:** Hair-related keratins and epithelial keratins expression in the nail bed of onychopapilloma.

Onychopapilloma	HK31	HK34	HK85	HK86	HK75	K6/K16
1	+	–	–	–	+	+
2	+	–	–	–	+	+
3	+	–	–	–	+	+
4	+	–	–	–	+	+
5	+	–	–	–	+	+
6	+	–	–	–	+	+
7	+	–	–	–	+	+
8	+	–	–	–	+	+
9	+	–	–	–	+	+
10	+	–	–	–	+	+
11	+	–	–	–	+	+

## Discussion

Baran and Perrin first reported four subungual keratosis cases with multinucleated cell aggregation according to pathological morphology and named the condition “local multinucleated distal keratosis” in 1995 (1). In 2000, they reported another 14 cases with longitudinal erythronychia and suggested that the term “local multinucleated distal keratosis” should be replaced by “onychopapilloma” (2). Onychopapilloma usually occurs in adults and most commonly in the thumb (2).

Clinically, onychopapilloma typically presents as longitudinal erythronychia with subungual hyperkeratosis. It can also present as longitudinal melanonychia, longitudinal leukonychia, splinter hemorrhages or onycholysis. Although the longitudinal streak can extend from the free edge of the nail plate to the lunula, this does not suggest that onychopapilloma originates from the nail matrix. Halteh suggested that longitudinal erythronychia may be due to the tumor compression of the nail bed causing local nail plate thinning and the formation of the lower erythema (10). The longitudinal leukonychia may be caused by the photorefraction change and fibrosis of the nail bed epithelium. And longitudinal melanonychia may be associated with the activation of melanocytes stimulated by the tumor. Therefore, the specific location and origin of onychopapilloma cannot be determined only through clinical manifestations. The keratotic mass under the nail plate corresponds to the position of the longitudinal streak. The dermoscopic examination can more clearly show the asymmetric keratotic mass under the nail plate with excessive lamellar keratinization. Splinter hemorrhages were observed in some patients, presenting continuous or discontinuous, single or multiple. Therefore, the dermoscopic examination is very important in the diagnosis of onychopapilloma.

In previous studies of onychopapilloma, it was recognized as a benign neoplasm originating from the distal nail matrix. However, the etiology and pathogenesis of onychopapilloma have not reached a consensus yet. Anatomically, the nail bed is different from the nail matrix, and there is no transformation between the two. We hope to determine whether onychopapilloma originates from the nail bed, nail matrix, or both.

On histological grounds, onychopapilloma is characterized by acanthosis and papillomatosis within the nail bed and abundant eosinophilic cytoplasm in the upper cell layers of the nail bed epithelium (matrix metaplasia) (2–4, 10–12). Sometimes, matrix metaplasia cannot be observed because of the surgical method or the quality of the specimen. Thus, in other disease premises, the diagnosis could be made histologically based on acanthosis and papillomatosis within the nail bed epithelium. Additionally, histopathology showed that onychopapilloma was a mixed growth acanthoma that can secrete large amounts of eosinophilic keratin (13). It is associated with subungual hyperkeratosis or subungual keratotic mass. Histologically, hyperkeratosis and parakeratosis are helpful in diagnosis.

The differential diagnosis of onychopapilloma mainly includes Bowen’s disease, glomus tumor, Darier disease, and melanoma. There is no distal subungual keratosis in Bowen’s disease, and the pathological features are obvious, with typical anomalous epidermal cells and dyskeratosis cells. Glomus tumor patients usually have severe pain and no distal subungual hyperkeratosis. Longitudinal erythronychia is also a typical manifestation of Darier disease, but it usually involves more than one finger and is often accompanied by longitudinal leukonychia. When onychopapilloma presents as longitudinal melanonychia, the pathological examination should be performed to eliminate melanoma. Usually, clinical manifestation, dermatoscopy, and histopathological examination are sufficient to confirm the diagnosis of onychopapilloma. However, because of the particularity of the histology of the nail unit, it is challenging to distinguish onychopapilloma from other onycho-tumors, such as onychocytic matricoma and onychomatricoma. In these circumstances, the immunohistochemical feature is helpful.

We studied 11 cases of onychopapilloma and confirmed that all histopathologic changes of onychopapilloma were seen in the nail bed rather than the nail matrix. Therefore, we preliminarily conjecture that the onychopapilloma originates from the nail bed.

In recent studies, the expression profiles of hair-related keratins and epithelial keratins have been determined in the standard nail unit (6–9). Hair-related keratins, including HK31, HK34, HK85, and HK86, were specifically expressed in the nail matrix and nail plate but not in the standard nail bed or nail fold. The expression of HK75 was supposed to be limited in the superbasal nail bed epithelium, and K6/K16 was expressed both in the nail bed and eponychium. The special expression pattern of hair-related keratins and epithelial keratins in the nail unit could be used to identify the differentiation of nail matrix epithelium and nail bed epithelium. Thus, we performed an immunohistochemical study to clarify the origin of onychopapilloma. There was no significant difference between the proximal and distal parts of the tumor (Figure 4), so we selected the more prominent portion of the tumor at the distal nail bed. The results showed that HK75 and K6/K16 were strongly expressed positively in the nail bed, especially papillomatosis with acanthosis of the nail bed epithelium. In contrast, hair-related keratins, including HK34, HK85, and HK86, showed negative expression in onychopapilloma. This indicated that onychopapilloma is a benign tumor of the nail bed rather than the nail matrix. Subungual hyperkeratosis was formed by keratin accumulation produced by neoplastic hyperplasia of the nail bed epithelium. However, the expression of HK31 requires an explanation. HK31 was supposed to be expressed only in the nail matrix. Our study showed that it was expressed both in the standard nail matrix and the involved nail bed epithelium, especially in the superficial layer of the nail bed (matrix metaplasia). Previously, the structure of the nail unit is usually recognized as an equivalent of the follicle. In the hair matrix, HK85 is the marker protein that showed persistent positive expression. HK31 is early cortex keratin in the inferior part of the hair cortex, the expression of which disappears at an early stage. While HK86 and HK34 are late cortex keratins in the upper part of the hair cortex, which express at the late stages (14). By analogy with hair follicles, we suspect that the expression of HK31 may be associated with matrix metaplasia of the nail bed epithelium, which leads to an increased ability of the nail bed to produce keratin.

To our knowledge, the current study is the first clinical research to explore the origin of onychopapilloma by immunohistochemistry to date. From our result, the specific expression of HK31, the positive expression of “nail bed-related keratin” (HK75, K6/K16) and the negative expression of “nail matrix-related keratin” (HK34, HK85, and HK86) could be the immunohistochemical characteristic of onychopapilloma. Based on the specific expression of HK31 in the nail bed, we speculate that HK31 can also be used as a marker for the differential diagnosis of onychopapilloma and other nail bed tumors.

## Conclusion

Our study suggests that onychopapilloma may originate from the nail bed epithelium. The histopathological characteristics of onychopapilloma include papillomatosis, acanthosis and matrix metaplasia of the nail bed epithelium rather than the nail matrix. In immunohistochemistry, onychopapilloma showed positive expression of HK31 and “nail bed-related keratin,” with negative expression of other “nail matrix-related keratin,” which may provide a new approach for the differential diagnosis of onychopapilloma.

## Data availability statement

The raw data supporting the conclusions of this article will be made available by the authors, without undue reservation.

## Ethics statement

The studies involving human participants were reviewed and approved by the Ethics Committee of the First Affiliated Hospital with Nanjing Medical University, Nanjing, China. The patients/participants provided their written informed consent to participate in this study.

## Author contributions

DW: conceptualization, funding acquisition, and resources. ML, FL, XW, ZL, HW, and YZ: investigation. ML, FL, and XW: formal analysis. ML: writing—original draft preparation. FL and XW: writing—review and editing. FL and DW: supervision. ML and DW: project administration. All authors contributed to the article and approved the submitted version.
